# Health Tract, No. 33

**Published:** 1862-02

**Authors:** 


### HEALTH TRACT, No. 33.

---

### DYSPEPSIA.

Dyspepsia is the inability of the stomach to prepare from the food eaten the nourishment requisite to sustain the body, and to supply it with pure blood, which, in its impure, unnatural condition, is sent to every fiber of the system; hence there is not a square inch of the body which is not liable to be affected with uneasiness or actual pain, and that portion will suffer most which has been previously weakened, or diseased, or injured in any way. Hence among a dozen dyspeptics, no two will have the same predominant symptoms, either in nature or locality; and as these persons differ further in age, sex, temperament, constitution, occupation, and habits of mind and body, it is the hight of absurdity to treat any two dyspeptics precisely alike; hence the failure to cure in many curable cases.

Dyspeptics of high mental power and of a bilious temperament, are subject to sick-headache; those who are fat and phlegmatic, have constipation and cold feet; while the thin and nervous have horrible neuralgies, which make of life a continued martyrdom, or they are abandoned to forebodings so gloomy, and even fearful sometimes, as to eat out all the joy of life, and make death a longed-for event. Some dyspeptics are wonderfully forgetful; others have such an irritability of temper as to render companionship with them, even for a few hours, painful, while there is such a remarkable incapacity of mental concentration, of fixedness of purpose, that it is impossible to secure any connected effort for recovery.

There are some general principles of cure applicable to all, and which will seldom fail of high advantages.

1. The entire body should be washed once a week with soap, hot water, and a stiff brush.

2. Wear woolen next the skin the year round, during the daytime only.

3. By means of ripe fruits and berries, coarse bread, and other coarse food, keep the bowels acting freely once in every twenty-four hours.

4. Under all circumstances, keep the feet always clean, dry, and warm.

5. It is most indispensable to have the fullest plenty of sound, regular, connected, and refreshing sleep in a clean, light, well-aired chamber, with windows facing the sun.

6. Spend two or three hours of every forenoon, and one or two of every afternoon, rain or shine, in the open air, in some form of interesting, exhilarating, and unwearying exercise—walking, with a cheering and entertaining companion is the very best.

7. Eat at regular times, and always slowly.

8. That food is best for each which is most relished, and is followed by the least discomfort. What may have benefited or injured one, is no rule for another. This eighth item is of universal application.

9. Take but a teacupful of any kind of drink at one meal, and let that be hot.

10. Confine yourself to coarse bread of corn, rye, or wheat—to ripe, fresh, perfect fruits and berries, in their natural state—and to fresh lean meats, broiled or roasted, as meat is easier of digestion than vegetables. Milk, gravies, pastries, heavy hot bread, farinas, starches, and greasy food in general, aggravate dyspepsia by their constipating tendencies.

11. It is better to eat at regular times as often as hungry, but so little at once, as to occasion no discomfort whatever.

12. Constantly aim to divert the mind from the bodily condition, in pleasant ways; this is half he cure in many cases.

